# Mechanobiological feedback loops and quantitative decision thresholds in organ fibrosis: Translational principles for antifibrotic therapy

**DOI:** 10.1002/ccs3.70111

**Published:** 2026-09-01

**Authors:** Amedeo Lonardo, Ralf Weiskirchen

**Affiliations:** ^1^ Independent Researcher Modena Italy; ^2^ Azienda Ospedaliero‐Universitaria Modena Italy; ^3^ Institute of Molecular Pathobiochemistry, Experimental Gene Therapy, and Clinical Chemistry (IFMPEGKC) RWTH University Hospital Aachen Aachen Germany

**Keywords:** epithelial‐mesenchymal transition, extracellular matrix remodeling, extracellular vesicles, fibrosis, mechanotransduction, signaling pathways, YAP/TAZ signaling

## Abstract

Multiorgan fibrosis is a complex, maladaptive response to chronic injury, where the extracellular matrix (ECM) shifts from serving merely as a structural framework to becoming an active, sustained contributor to disease progression. We propose a unifying mechanobiological framework in which chronic injury promotes fibroblast activation and ECM remodeling, whereas the stiffened and remodeled ECM feeds back through integrins, mechanosensitive ion channels, collagen receptors, and Hippo‐YAP/TAZ signaling to sustain fibrogenesis. Regulation of this process is governed by bidirectional feedback between cellular signaling and the matrix's physical properties across organs such as the lung, heart, liver, and kidney. This narrative review article presents recent advances in understanding mechanisms of cell–cell and ECM communication, paratensile signaling, mechanical feedback loops, cellular crosstalk and secretomes, quantitative therapeutic thresholds, and translational strategies for tissue remodeling. These developments collectively offer promising insights for the development of novel pharmacological agents targeting mechanotransduction pathways, ECM normalization, and cell‐specific sensitization. We further define quantitative thresholds as diagnostic, action, and response thresholds that may support clinical decisions such as referral, treatment initiation, therapeutic monitoring, and clinical‐trial enrichment. Finally, we provide specific details regarding the treatment of liver fibrosis before concluding with research agenda.

## Graphical abstract

Multiorgan fibrosis arises when chronic injury activates fibroblasts and remodels extracellular matrix, whose increasing stiffness sustains disease through mechanotransduction pathways. Bidirectional cellular‐matrix feedback operates across lung, heart, liver, and kidney. Emerging diagnostic, action, and response thresholds may guide referral, treatment, monitoring, trial enrichment, and therapies targeting matrix normalization and cell‐specific signaling.

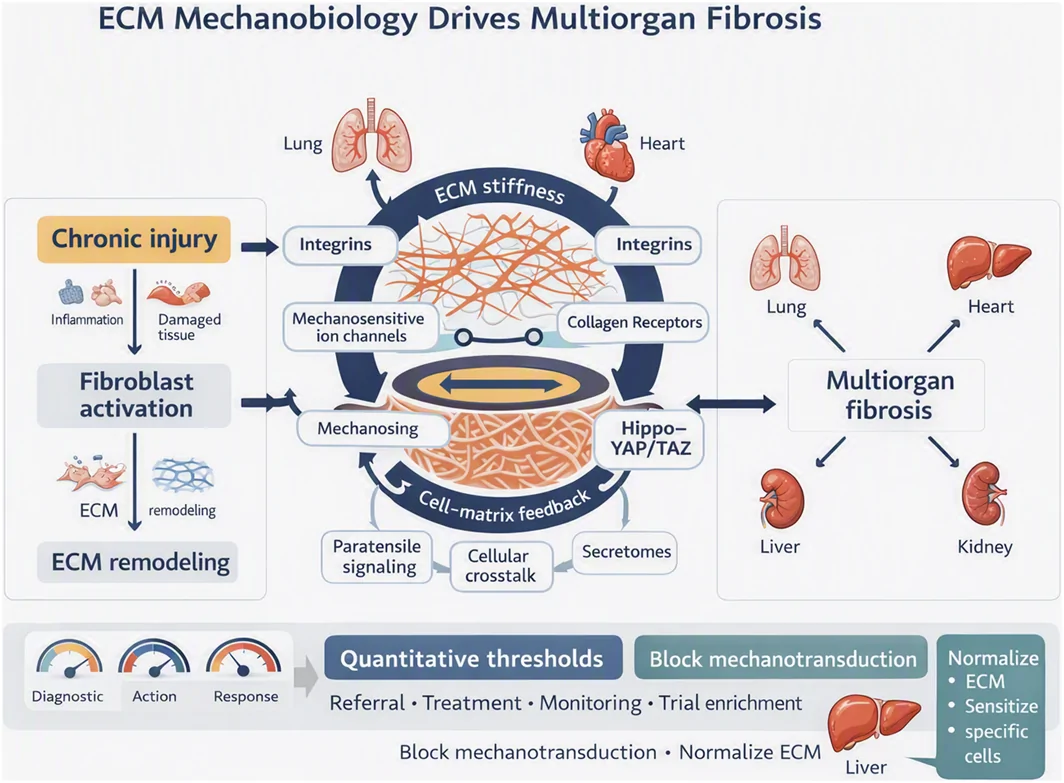

## 1 INTRODUCTION

### 1.1 Definitions and pathobiology

Fibrosis is characterized by excessive deposition of extracellular matrix (ECM) components, including fibrillar collagens, fibronectin, and proteoglycan, produced primarily by activated myofibroblasts (MFs) during chronic tissues injury, which is further associated with inflammation, tissue damage, and faulty repair in tissues and organs.^1^ Multiorgan fibrosis is a complex, maladaptive response to chronic injury where the ECM transitions from a passive scaffold to an active, self‐sustaining driver of disease.^2^ This progression is governed by a bidirectional feedback loop between cellular signaling and physical matrix properties across organs such as the lung, heart, liver, kidneys, and skin.^1^, ^3^ Fibrosis, whose clinical manifestations vary by organ, is a serious condition impacting millions worldwide. It reduces vital organ function and raises the risks of end‐stage disease and cancer, thereby contributing to disability or death.^1^, ^4^

### 1.2 Burden

Multiorgan fibrosis, which encompasses a range of clinical conditions including lung fibrosis, chronic cardiac and renal diseases, accounted for 16.5% of global deaths in 1990. This proportion consistently increased, reaching 17.8% in 2019.^5^ Several factors contribute to the substantial burden, including hospitalization expenses, increased costs associated with disease progression, and transplantation expenses. Hospital stays for idiopathic pulmonary fibrosis (IPF) often cost more than $16,000 per admission.^6^ Similarly, chronic fibrosing liver disease results in a global economic burden reaching $2.649 trillion between 2021 and 2050.^7^

### 1.3 Aim

Here, we outline the molecular mechanisms behind cell–cell and ECM interactions, paratensile signaling, mechanical feedback, cellular communication, therapeutic thresholds, and translational approaches for tissue remodeling. Although our discussion does not focus on individual organ systems, it includes examples of IPF, fibrosis in the kidneys, liver, and skin. This review provides valuable insights for developing novel therapeutics aimed at mechanotransduction, ECM normalization, and tailored cellular responses.

### 1.4 Conceptual framework: The mechanobiological fibrosis loop

Fibrosis can be understood as a self‐reinforcing mechanobiological loop in which chronic injury activates inflammatory, epithelial, endothelial, immune, and mesenchymal cell programs that converge on fibroblast activation and ECM remodeling. Activated fibroblasts and MFs deposit collagen, fibronectin, proteoglycans, and other ECM components, which progressively alter matrix density, architecture, crosslinking, and stiffness. The remodeled matrix is then sensed by cells through collagen receptors, integrins, focal adhesions, mechanosensitive ion channels, and cytoskeletal tension pathways, leading to activation of intracellular signaling programs such as calcium signaling, FAK‐Rho‐actomyosin signaling, transforming growth factor‐β (TGF‐β) activation, and Hippo‐YAP/TAZ‐dependent transcription. These pathways further increase fibroblast contractility, survival, proliferation, and matrix production, thereby closing a pathological feedback loop between cellular signaling and the physical properties of the ECM. Within this framework, paratensile signaling represents a mechanism by which contractile cells transmit mechanical information through collagen fibers to neighboring or distant fibroblasts. Macrophage–fibroblast interactions, extracellular vesicles (EVs), and partial epithelial or endothelial mesenchymal transitions should be viewed as amplifiers or modulators of this loop rather than as isolated mechanisms. The translational goal of mechanobiology is therefore to identify measurable points within this loop that can be used for diagnosis, risk stratification, treatment selection, and monitoring of antifibrotic response.

## 2 METHOD OF BIBLIOGRAPHIC RESEARCH

For this narrative review, we undertook a structured search of PubMed/MEDLINE, Scopus, and Web of Science to identify recent advances in the mechanobiology of fibrosis and their translational implications. The search strategy combined terms related to fibrosis and mechanobiology, including “fibrosis,” “extracellular matrix,” “mechanotransduction,” “paratensile signaling,” “Hippo signaling,” “YAP/TAZ,” “fibroblast activation,” “EVs,” “epithelial‐mesenchymal transition,” “endothelial‐mesenchymal transition,” “imaging biomarkers,” and “antifibrotic therapy.” Boolean operators were used to identify studies spanning molecular mechanisms and translational or clinical applications.

We prioritized peer‐reviewed reports published within the last 10–15 years, particularly high‐impact original studies, systematic reviews, and major translational investigations linking mechanistic findings to therapeutic development. Earlier landmark studies were retained when needed to establish the historical or conceptual basis of mechanotransduction and matrix‐mediated signaling.

We selected studies addressing cell–cell and cell–extracellular matrix communication, mechanosensitive signaling, and emerging antifibrotic strategies across the liver, lung, heart, kidney, and skin. To retain a translational focus on major systemic fibrotic diseases, we excluded research confined to localized or rare entities, such as ocular or retroperitoneal fibrosis.

We also reviewed clinical studies of imaging‐based fibrosis quantification, digital pathology, and pharmacological interventions to connect mechanobiological discoveries with clinical practice. Reference lists of key articles were screened for additional eligible reports. The final literature set was curated to balance molecular mechanisms, quantitative diagnostic approaches, and emerging strategies to disrupt the pathological feedback loop between cells and the ECM.

## 3 MECHANISMS OF CELL–CELL AND ECM SIGNALING

The mechanisms discussed below are best interpreted as interconnected components of a single mechanobiological circuit rather than as independent pathways. Collagen remodeling and ECM stiffening generate mechanical cues that are detected by collagen receptors such as discoidin domain receptor family, member 2 (DDR2), adhesion receptors such as integrins, mechanosensitive ion channels such as Piezo1, and focal adhesion‐cytoskeletal complexes. These upstream sensors activate calcium signaling, actomyosin contractility, focal adhesion signaling, latent TGF‐β activation, and Hippo‐YAP/TAZ‐dependent transcription. The resulting transcriptional programs promote fibroblast proliferation, contractility, survival, and ECM synthesis. Macrophages, EVs, epithelial cells, endothelial cells (ECs), and perivascular cells modify this circuit by supplying cytokines, growth factors, microRNAs, matrix‐remodeling enzymes, and additional mesenchymal‐like cells. Thus, progressive fibrosis emerges from the integration of mechanical force transmission, biochemical signaling, and multicellular communication.

The progression of fibrosis critically depends on the coordinated interaction between biochemical and biomechanical signals that initiate and maintain the activation of effector cells.

### 3.1 Paratensile signaling

Paratensile signaling defines a newly recognized and incompletely characterized mechanism of mechanical communication whereby cells convey long‐range mechanical signals via collagen fibers, independently of traditional chemical paracrine signaling pathways. Understanding how cells sense and respond to mechanical signals from ECM and blood flow is a complex and important research challenge.

**FIGURE 1:**
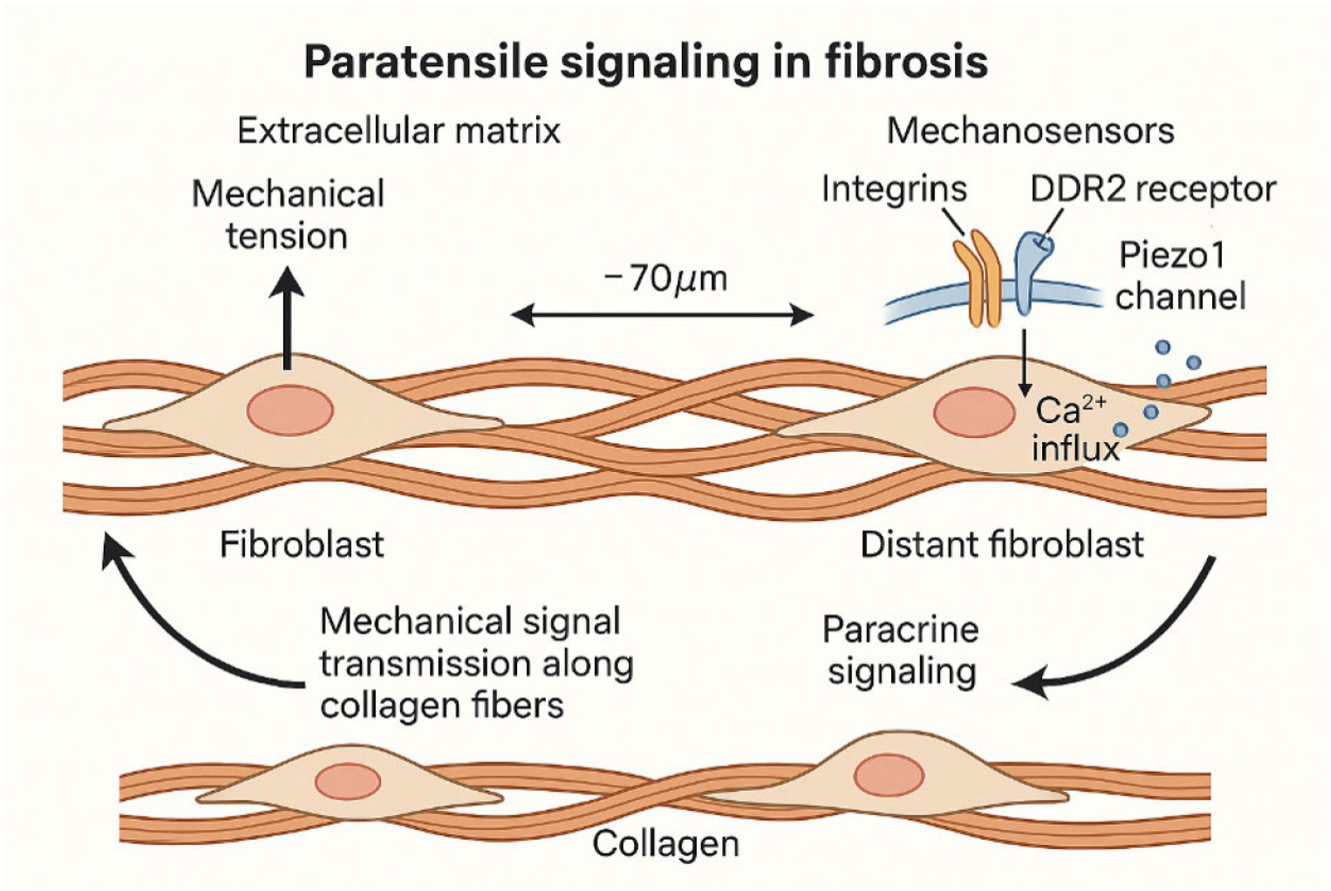
Paratensile signaling in fibrotic tissue microenvironments. Paratensile signaling is a type of long‐range mechanical communication between cells facilitated by the ECM. When an activated fibroblast generates contractile forces, these forces are transmitted along aligned collagen fibers, allowing mechanical signals to travel through the matrix and activate distant fibroblasts. This process occurs independently of classical paracrine signaling and can operate over distances of up to approximately 70 μm. Mechanosensitive receptors such as discoidin domain receptor 2, integrins, and the mechanosensitive ion channel Piezo1 play a role in detecting matrix deformation and initiating intracellular calcium signaling in nearby cells. Through this mechanism, localized mechanical stress can spread throughout the ECM network, promoting synchronized fibroblast activation and facilitating the spatial expansion of fibrosis. This figure was created using copilot and subsequently reviewed and edited by the authors. ECM, extracellular matrix.

Liu et al.^8^ were the first to propose the concept of paratensile signaling. This process rapidly transmits signals through collagen fibers, activating fibroblasts within 1 s over distances up to 70 μm. DDR2 and integrin pathways mediate this, relying on calcium and the mechanosensitive Piezo1 ion channel. These researchers used fibroblast growth models and mathematical analysis to show that paratensile signaling at the single‐cell level helps explain tissue fibrosis progression. Blocking paratensile signaling reduced fibroblast‐to‐myofibroblast conversion at fibrotic‐normal tissue boundaries, which provides insights into cell dynamics in fibrosis and suggests paratensile signaling as a potential antifibrotic therapy target (Figure 1).

Expanding this study, Long et al.^9^ identified mechanical hallmarks of fibrotic microenvironments as both consequences and drivers of fibrosis progression. Recent findings highlight how intracellular mechanotransduction pathways adapt to ECM changes and abnormal hemodynamics, such as low shear stress, excessive stretch, and high pressure, which are key biomechanical features of fibrosis in organs like the liver, lung, and heart. The authors define mechanical communication in cell–ECM interactions, hemodynamics, and paratensile signaling during fibrosis growth. They also review engineering platforms for disease modeling to identify and predict mechanobiological targets that reduce fibrosis progression.

### 3.2 Mechanical feedback loops

**FIGURE 2:**
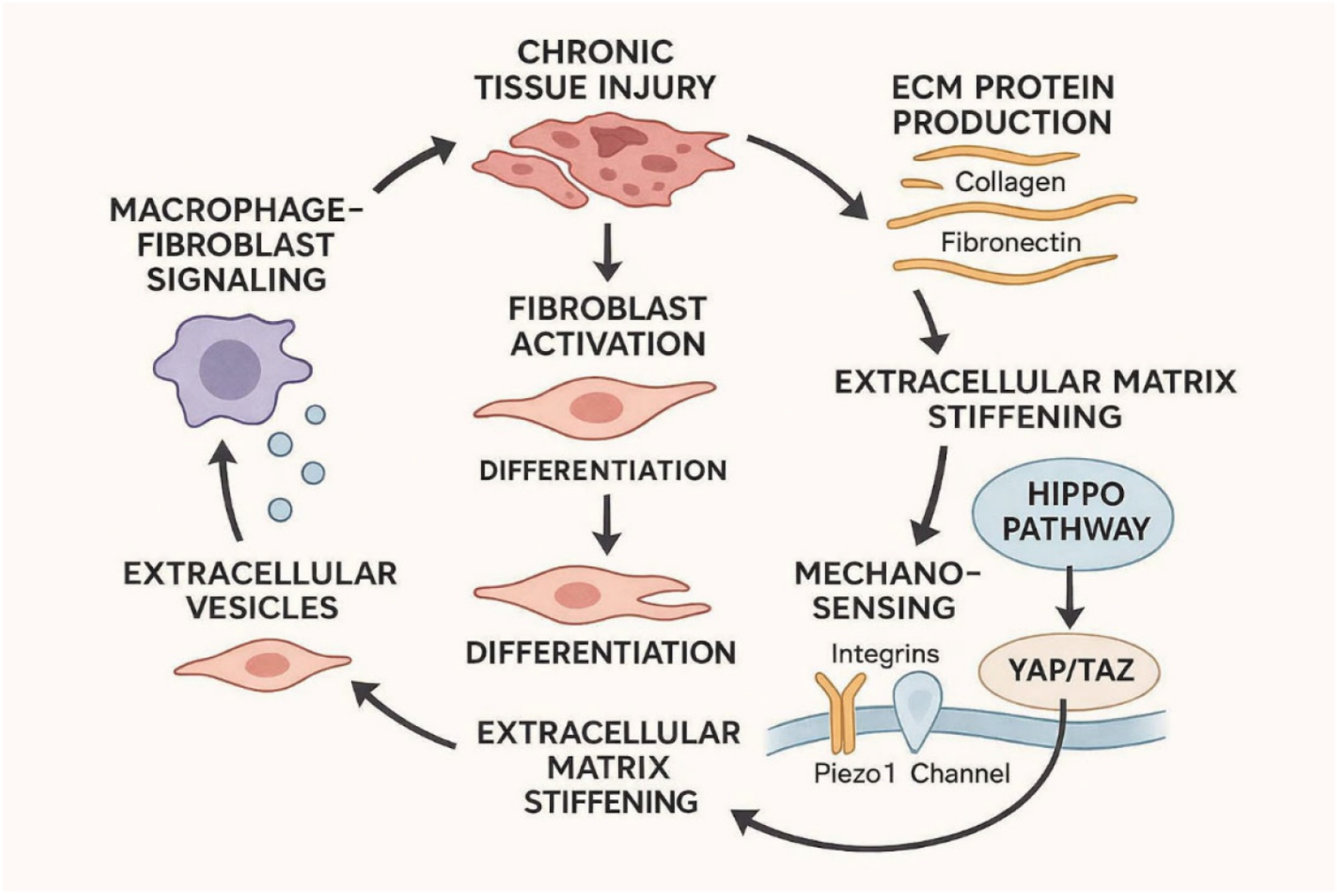
Mechanobiological feedback loop driving fibrosis progression. Chronic tissue injury initiates inflammatory responses that activate fibroblasts and promote their differentiation into contractile myofibroblasts. These cells produce excessive ECM components, including collagen and fibronectin, leading to progressive matrix accumulation and increased tissue stiffness. Mechanical changes in the ECM are sensed by cells through mechanosensitive structures such as integrins and ion channels including Piezo1, which activate intracellular signaling pathways such as the Hippo pathway and its transcriptional co‐activators such as YAP and TAZ. Nuclear YAP/TAZ signaling promotes gene expression programs that enhance fibroblast activation, proliferation, and further ECM synthesis. In parallel, macrophage–fibroblast interactions and extracellular vesicle‐mediated communication reinforce the profibrotic microenvironment. Together, these processes establish a self‐reinforcing feedback loop in which matrix stiffening and cellular activation mutually amplify each other, sustaining progressive fibrosis across multiple organs. This figure was created using copilot and subsequently reviewed and edited by the authors. ECM, extracellular matrix; TAZ, transcriptional coactivator with PDZ‐binding motif; YAP, yes‐associated protein.

When the ECM stiffens, fibroblasts sense increased tension via integrin receptors, activating the Hippo signaling pathway, an evolutionary conserved regulator of organ size, tissue repair, and human development^10^ and its effectors yes‐associated protein (YAP)/transcriptional coactivator with PDZ‐binding motif (YAP/TAZ) (Figure 2). This mechanical sensing triggers MF differentiation and further ECM deposition, creating a “vicious cycle” of stiffening. Hippo signaling inhibits YAP/TAZ interactions with transcription factors such as TEA domain transcription factor (TEAD) and Smad, impacting gene expression tied to cell growth and survival. Mouse studies show YAP/TAZ is crucial for liver development, regeneration, homeostasis, and tumorigenesis.^11^ YAP/TAZ activation—initially thought to be active only in cholangiocytes—occurs in various hepatic cells during chronic liver disease. Dysregulation of YAP/TAZ is central to metabolic dysfunction (MASLD) progression, leading to hepatocyte dysfunction, inflammation, and fibrosis highlighting the multifaceted impact of YAP/TAZ on liver physiology and pathology.^12^

Early MASLD suppresses tumor initiation in mouse liver cancer models by activating YAP/TAZ‐mediated cell competition, which reveals a surprising tumor‐suppressive effect, emphasizing the role of noncell autonomous mechanisms. Given widespread Hippo‐YAP/TAZ deregulation, similar processes may occur in other tissues.^13^ Liu et al. have demonstrated that adipose‐derived mesenchymal stem cells (ADMSCs) suppress hepatic stellate cell (HSC) activation through the regulation of YAP/TAZ, thereby facilitating functional recovery following liver fibrosis.^14^ These findings support future research into the specific mechanisms by which ADMSCs mitigate liver fibrosis.

In addition to the liver, the Hippo signaling pathway is involved in renal fibrosis, diabetic nephropathy, and renal cancer.^10^ Other organ systems in which the YAP/TAZ effectors are implicated comprise lung, heart, and skin.^4^

### 3.3 Cellular crosstalk and secretomes

#### 3.3.1 Macrophage–fibroblast axis

The macrophage–fibroblast axis involves bidirectional signaling in which activated fibroblasts shape macrophage recruitment and phenotype, whereas profibrotic macrophage states promote fibroblast activation, survival, contractility, and matrix production through cytokines, growth factors, lipid mediators, and matrix‐remodeling enzymes. Although macrophages can contribute to ECM remodeling and may express selected matrix components in specific contexts, they should not be presented as a universal major source of collagen or fibronectin across all fibrotic organs.

Investigating macrophage–fibroblast interactions uncovers crucial targets for treating fibrotic diseases. The accumulation of ECM and tissue stiffness arises from the intricate relationship between these cells, potentially leading to organ dysfunction. Macrophages influence fibroblasts through cytokines and growth factors, directing their growth and specialization, whereas active fibroblasts generate ECM and influence macrophage behavior in return.^15^ This reciprocal communication sustains fibrosis and contributes to tissue dysfunction.

#### 3.3.2 Extracellular vesicles

EVs transport profibrotic signals among epithelial, endothelial, and immune cells within the chronic wound‐healing environment, playing key roles in fibrosis. For example, EVs from chronic wounds promote profibrotic signals, whereas those from stem cells and healthy cells exhibit antifibrotic properties, offering new therapeutic possibilities. EVs present in body fluids may also serve as minimally invasive biomarkers for accurately monitoring fibrosis progression or regression.^1^ Additionally, EVs found in human milk are one of the mechanisms involved in the hepatoprotective activity of breastfeeding.^16^

#### 3.3.3 Mesenchymal transitions

**FIGURE 3:**
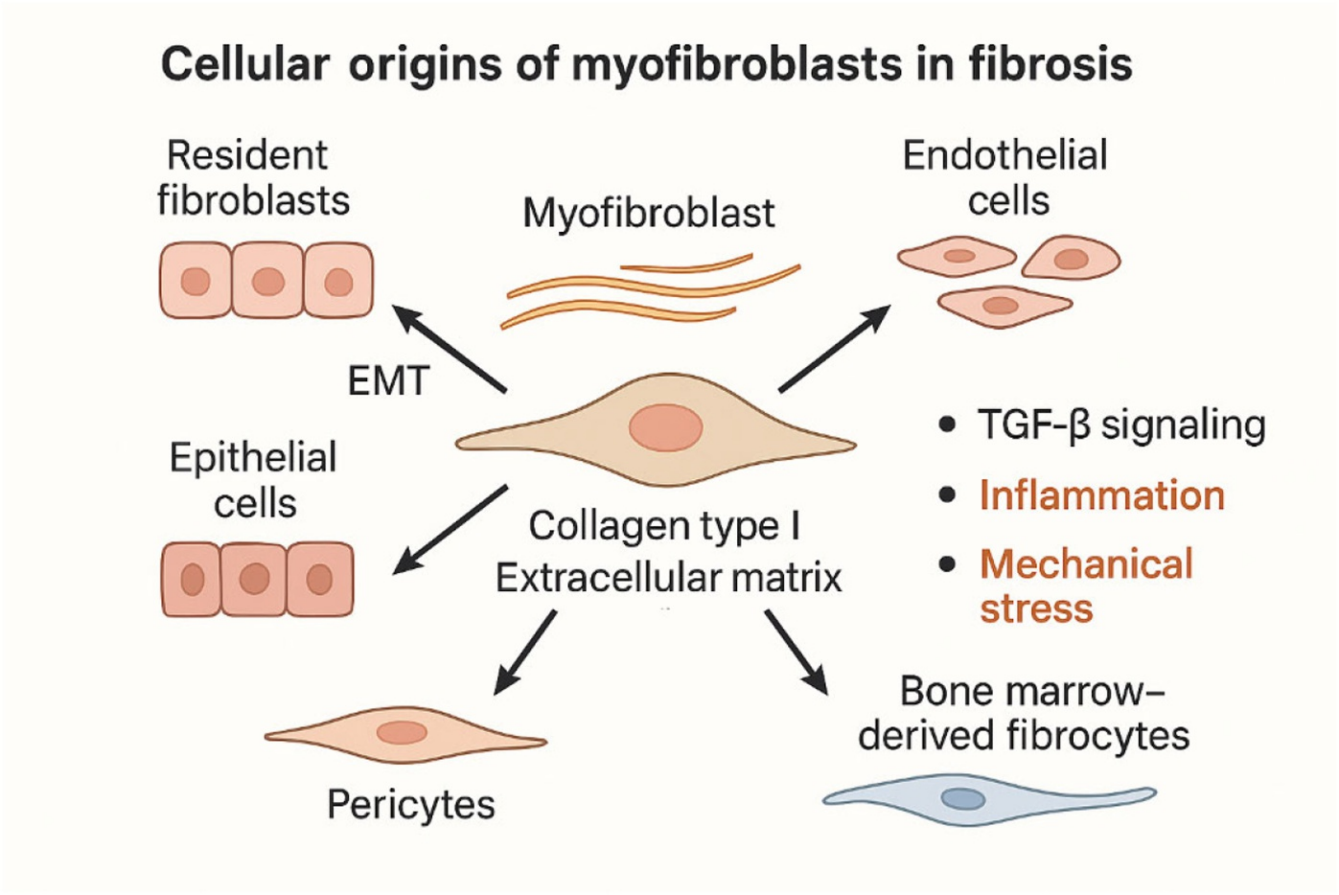
Cellular sources of MFs in fibrotic tissues. MFs, the primary effector cells responsible for excessive ECM deposition in fibrosis, arise from multiple cellular origins. Resident fibroblasts represent a major source, undergoing activation in response to inflammatory and mechanical signals. Additional contributors include bone marrow‐derived fibrocytes, epithelial cells undergoing epithelial‐to‐mesenchymal transition, endothelial cells undergoing endothelial‐to‐mesenchymal transition, and perivascular cells such as pericytes. These cellular transitions are driven by profibrotic stimuli including transforming growth factor‐β, inflammatory cytokines, and mechanical stress within the ECM. Regardless of origin, activated MFs acquire a contractile phenotype characterized by the expression of α‐smooth muscle actin and enhanced production of collagen type I and other matrix components, contributing to progressive ECM accumulation and tissue stiffening. This figure was created using copilot and subsequently reviewed and edited by the authors. ECM, extracellular matrix; MFs, myofibroblasts.

MFs, the primary producers of collagen I, can originate from resident fibroblasts, bone marrow‐derived fibrocytes, or through endothelial‐to‐mesenchymal transition (EndoMT) and epithelial‐mesenchymal transition (EMT) (Figure 3). EndoMT is a multifaceted biological process during which ECs undergo transformation into cells exhibiting a mesenchymal phenotype. This cell transformation involves the adoption of characteristic mesenchymal cell morphology, acquisition of cellular motility, downregulation of EC‐specific markers, and initiation of mesenchymal cell‐specific gene expression as well as the synthesis of fibrillar collagens.^17^

TGF‐β signaling is a major inducer of EndoMT‐like programs in fibrotic disorders, but the extent and consequences of EndoMT are modulated by inflammatory mediators, mechanical stress, endothelial subtype, tissue microenvironment, and disease stage. Furthermore, additional, yet unidentified, regulatory pathways may participate in EndMT depending on specific physiological conditions and cellular environments.^18^

A study using single‐cell RNA sequencing, lineage tracing, and colocalization in a mouse liver fibrosis model found that although some liver sinusoidal endothelial cells (LSECs) become MFs, most undergo capillarization and transition to mesenchymal‐like cells.^17^ This phenotypic shift resembles partial EndMT and results in LSECs serving as significant contributors of ECM within hepatic sinusoids.^17^

Although the molecular pathogenesis of fibrosis varies by organ and tissue, TGF‐β and inflammation are consistently key triggers in fibrogenic processes.^19^ TGF‐β and other cytokines regulate EMT, a process that may contribute to the expansion of mesenchymal cell populations during fibrotic remodeling. This is often termed “Type 2 EMT,” which occurs during repair‐associated processes when chronic inflammation causes an unresolved, pathological wound‐healing response.^20^

EMT is a context‐dependent process that may contribute to fibrogenesis by promoting epithelial plasticity, inflammatory signaling, and partial acquisition of mesenchymal features, but its quantitative contribution to the MF pool varies across organs, disease models, and stages of injury.^20^ Advancing knowledge of the interactions between fibrosis and EMT transition could facilitate the creation of comprehensive antifibrotic treatments.

## 4 QUANTITATIVE THERAPEUTIC THRESHOLDS

Quantitative thresholds in fibrosis should not be understood only as diagnostic cutoffs, but as operational decision points that connect biological remodeling to clinical action (Table 1). At least three types of thresholds can be distinguished. First, diagnostic thresholds identify when ECM accumulation or tissue stiffness exceeds the range expected for physiological repair. Second, action thresholds indicate when a patient should undergo referral, intensified monitoring, etiological treatment, antifibrotic therapy, or inclusion in a clinical trial. Third, response thresholds define the magnitude of structural, mechanical, biochemical, or functional improvement that is likely to represent meaningful disease modification rather than measurement variability. This framework links mechanobiology to clinical translation because it transforms continuous changes in ECM structure, stiffness, and cellular activation into measurable criteria for intervention and follow‐up.

**TABLE 1:** From quantitative fibrosis measurement to clinical decision‐making.

Domain	Quantitative measure	Example threshold concept	Clinical use	Limitation
Cardiac fibrosis	LGE‐CMR signal threshold versus reference myocardium	Lower signal thresholds may detect diffuse fibrosis, whereas higher thresholds may identify dense scar	Risk stratification, scar mapping, and monitoring structural remodeling	Thresholds vary by the acquisition method and disease context
Cardiac fibrosis	Image intensity ratio	Higher myocardial‐to‐blood‐pool signal suggests more advanced fibrosis or dense scar	Standardized fibrosis quantification and follow‐up	Requires technical standardization
Liver fibrosis	APRI, FIB‐4, and elastography	Low‐risk thresholds may rule out advanced fibrosis, whereas high‐risk thresholds may trigger specialist referral or therapy evaluation	Primary‐care triage, hepatology referral, treatment eligibility, and monitoring	Cutoffs vary by disease etiology, age, inflammation, and congestion
Liver fibrosis	qFibrosis or digital pathology	Continuous collagen‐architecture metrics may detect regression before ordinal stage change	Trial endpoints and early treatment‐response assessment	Requires biopsy tissue and validated image‐analysis pipelines
Kidney fibrosis	MRI‐based parameters and elastography approaches	Increasing stiffness or altered MRI parameters may indicate fibrotic remodeling	Noninvasive monitoring and trial enrichment	Still requires broader validation against outcomes
Lung fibrosis	HRCT‐based extent, quantitative CT, and pulmonary function trends	Progression thresholds can combine structural and functional change	Treatment initiation, progression monitoring, and trial endpoints	Imaging thresholds may not fully capture active fibroblast biology
Multiorgan fibrosis	Molecular imaging such as FAPI‐PET	Increased fibroblast activation signal may identify active remodeling before mature scar formation	Early intervention and target engagement	Clinical thresholds remain under development

Abbreviation: APRI, aspartate aminotransferase to platelet ratio index; FAPI‐PET, fibroblast activation protein inhibitor positron emission tomography; HRCT, high‐resolution computed tomography; LGE‐CMR, late gadolinium enhancement cardiovascular magnetic resonance; MRI, magnetic resonance imaging.

Quantifying fibrosis is crucial for establishing clinically relevant thresholds that differentiate between normal repair and abnormal remodeling, as well as for determining when medical intervention is needed.^21^ Traditional staging systems in many organs use ordinal histological scales to categorize fibrosis into distinct stages. Although useful in a clinical setting, these systems often do not fully capture the continuous nature of ECM accumulation and the dynamic processes involved in fibrotic progression or regression. As a result, there is a growing interest in quantitative methods that can detect subtle structural and functional changes in fibrotic tissues.

Imaging‐based metrics have become essential for noninvasively quantifying fibrosis. In cardiovascular medicine, late gadolinium enhancement (LGE) magnetic resonance imaging is a well‐established technique for visualizing myocardial fibrosis.^22^, ^23^ Quantification typically involves using signal intensity thresholds from the signal threshold versus reference myocardium method, where fibrotic tissue is identified by signal intensities exceeding a certain number of standard deviations above the mean signal of healthy myocardium.^24^ Studies have indicated that thresholds around three standard deviations above the reference mean are effective for detecting diffuse fibrotic areas, whereas higher thresholds around five standard deviations are better suited for identifying dense scar tissue, especially in cases of ablation‐induced lesions. Another quantitative approach is the image intensity ratio (IIR), which compares myocardial signal intensity to that of the blood pool. An IIR greater than approximately 1.20 is often considered indicative of fibrosis, whereas values exceeding about 1.32 are associated with dense scar formation.^25^, ^26^ LGE on cardiac magnetic resonance imaging has shown promise in predicting sudden death and guiding the use of preventive therapies in various cardiovascular conditions, including both ischemic and nonischemic cardiomyopathies.^23^

Beyond imaging, advances in digital pathology are transforming the evaluation of fibrosis at the microscopic level. Quantitative platforms such as qFibrosis utilize artificial intelligence‐assisted image analysis to create continuous numerical descriptors of collagen architecture, rather than relying solely on ordinal staging systems.^27^ These systems analyze spatial patterns of collagen fibers in tissue slides, including their distribution, connectivity, and thickness, allowing for the identification of subtle changes in fibrotic architecture that may indicate early therapeutic responses. Such methods have demonstrated their usefulness in liver disease, where portal and periportal collagen remodeling may occur before visible histological stage changes.^28^

**FIGURE 4:**
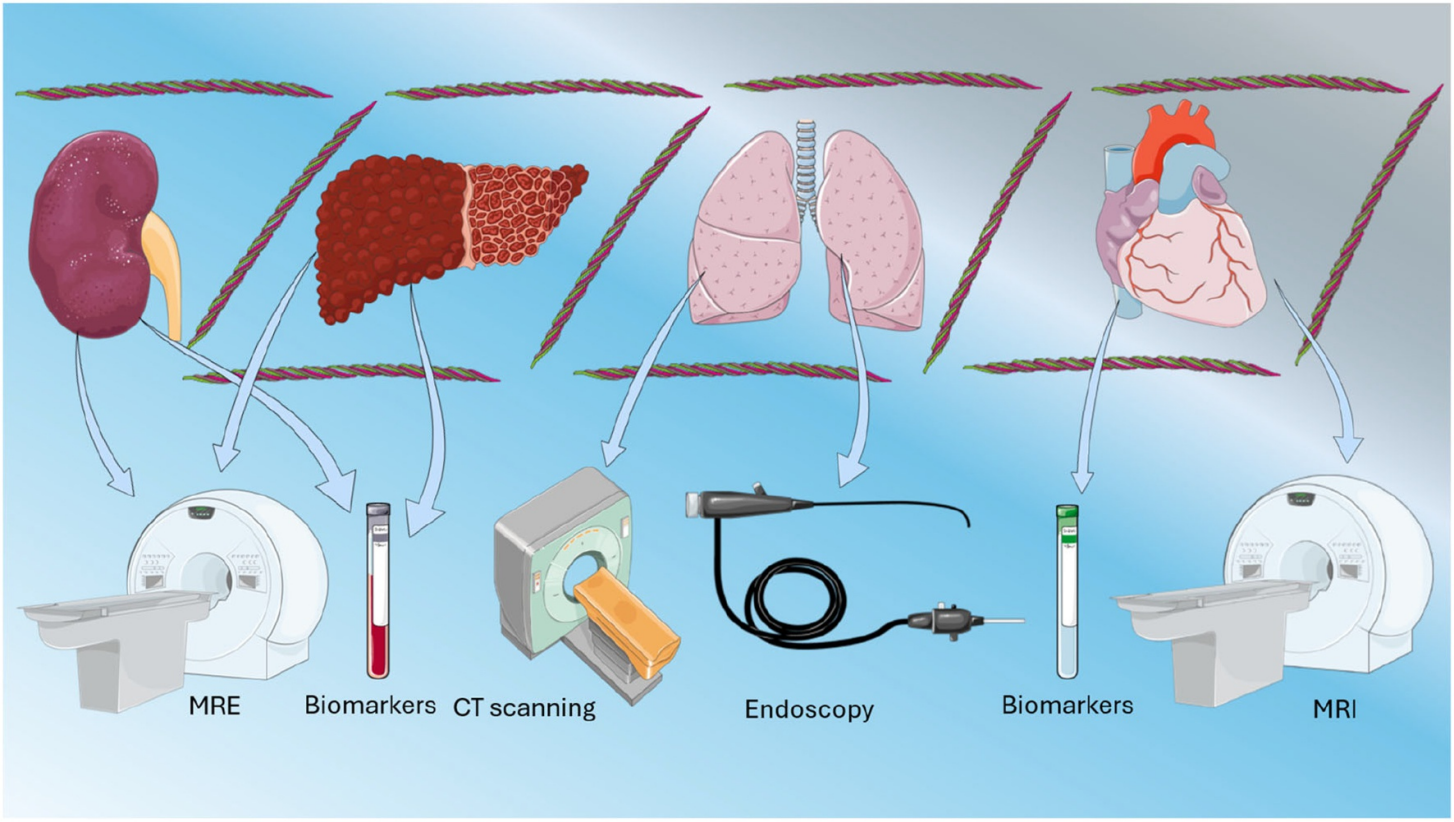
Assessing organ fibrosis in clinical practice noninvasively. Evidence shows that MRI parameters are strongly associated with renal histology variables indicating that MRI is an effective tool for assessing kidney fibrosis.^30^ Although liver biopsy has traditionally been considered the gold standard for diagnosing liver fibrosis, noninvasive methods, including simple scoring systems and validated panels combined with elastography, now make patient triage more efficient.^31^ These methods can be utilized in primary care and specialties such as diabetology or cardiology to identify individuals requiring hepatology referrals. Molecular markers are primarily used for research rather than daily clinical practice. The approach to diagnosing lung fibrosis emphasizes comprehensive clinical assessments and laboratory investigations to rule out secondary etiologies. High‐resolution computed tomography is a crucial early diagnostic modality, aiding in the identification of candidates for surgical lung biopsy to establish a definitive histopathological diagnosis.^32^ In the assessment of cardiac fibrosis, cardiac magnetic resonance imaging with T1 mapping and biomarkers plays a critical role, with natriuretic peptides providing diagnostic and prognostic value.^33^ MRE, magnetic resonance elastography; MRI, magnetic resonance imaging. The original illustration was created using Servier Medical ART and is licensed under the Creative Commons Attribution 4.0 International License (CC BY 4.0).

The concept of therapeutic thresholds arises from integrating quantitative measurements with clinical outcomes. Instead of defining fibrosis solely by histological stage, clinicians may increasingly rely on continuous biomarkers that indicate when matrix accumulation begins to impair organ function or when antifibrotic therapies achieve meaningful structural reversal. Identifying these thresholds could enable earlier intervention, improved monitoring of treatment efficacy, and more precise patient stratification in clinical trials. Ultimately, quantitative metrics linking ECM architecture, mechanical stiffness, and functional impairment may provide the foundation for personalized antifibrotic therapy. In the diagnosis of chronic hepatitis B, for example, a recent meta‐analysis that included nearly 20,000 records showed that the use of the aspartate aminotransferase to platelet ratio index or FIB‐4 scores and FibroScan, which are widely used to stage liver fibrosis and cirrhosis, is highly suitable to inform decisions on testing and treatment at a large scale. This takes into consideration a combination of factors, including diagnostic accuracy, availability, cost, and the number and potential consequences of false‐positive and false‐negative results.^29^ Figure 4 illustrates principles of noninvasive diagnosis of organ fibrosis in clinical practice.

The main translational challenge is to validate thresholds against outcomes that matter clinically, including organ function, hospitalization, transplantation, cancer risk, quality of life, and survival. For this reason, future antifibrotic trials should combine structural endpoints, such as collagen architecture or tissue stiffness, with functional endpoints, such as forced vital capacity (FVC), glomerular filtration rate, liver‐related events, arrhythmia risk, or heart‐failure progression. A threshold‐based approach may therefore improve patient selection, allow earlier intervention, and distinguish biological target engagement from clinically meaningful fibrosis reversal.

## 5 TRANSLATIONAL TARGETING FOR TISSUE REMODELING

The recognition that fibrosis is sustained by a reciprocal feedback loop between activated cells and the ECM has fundamentally reshaped the conceptual framework of antifibrotic therapy. In fibrotic tissues, activated fibroblasts and MFs continuously remodel the extracellular environment. In turn, the altered matrix influences cellular behavior through both mechanical and biochemical signals.^34^, ^35^ This bidirectional interaction generates a self‐reinforcing cycle in which increased ECM stiffness enhances mechanosensitive signaling pathways, further promoting fibroblast activation and additional matrix deposition. Consequently, therapeutic strategies increasingly aim to target not only inflammatory pathways but also the physical and biochemical properties of the ECM itself. Translational mechanobiology seeks to interrupt the pathological loop in which matrix stiffening activates fibroblasts, leading to increased production of ECM components such as collagen type I, fibronectin, and proteoglycans, ultimately resulting in progressive tissue rigidity and organ dysfunction.

The main mechanobiological targets currently considered for translational antifibrotic therapy are summarized in Table 2. These targets span ECM architecture, matrix crosslinking, cell–matrix adhesion, mechanosensitive ion channels, nuclear mechanotransduction, fibroblast persistence, senescence, immune amplification, and molecular imaging of fibroblast activation.

**TABLE 2:** Mechanobiological targets and translational therapeutic strategies in fibrosis.

Target level	Mechanism	Example target	Therapeutic concept
Matrix architecture	Collagen accumulation and fiber alignment	Collagen turnover pathways	Promote ECM remodeling
Matrix crosslinking	Increased collagen stiffness	LOX/LOXL enzymes	Reduce irreversible stiffening
Cell–matrix adhesion	Force transmission and latent TGF‐β activation	Integrins	Reduce mechanosignaling and TGF‐β activation
Ion‐channel mechanosensing	Mechanical calcium entry	Piezo1, TRPV4	Reduce stiffness‐induced fibroblast activation
Nuclear mechanotransduction	Mechanical gene transcription	YAP/TAZ‐TEAD	Reduce profibrotic transcription
Fibroblast persistence	Resistance to apoptosis	PI3K/AKT and BCL‐2 family	Restore myofibroblast clearance
Senescence	SASP‐driven inflammation and fibrosis	Senolytic pathways	Remove profibrotic senescent cells
Immune amplification	Macrophage–fibroblast signaling	CSF1R and TGF‐β‐related pathways	Reprogram profibrotic inflammation
Fibroblast activation imaging	Activated fibroblast detection	FAPI‐PET	Identify active remodeling before mature scar

Abbreviations: AKT, protein kinase B; ECM, extracellular matrix; FAPI‐PET, fibroblast activation protein inhibitor positron emission tomography; LOX, lysyl oxidase; LOXL, lysyl oxidase‐like proteins; TAZ, transcriptional coactivator with PDZ‐binding motif; TEAD, TEA domain transcription factor; TGF‐β, transforming growth factor‐β; YAP, yes‐associated protein.

One promising therapeutic direction involves mechano‐therapeutics that specifically target cellular mechanotransduction pathways.^34^, ^35^ Cells constantly sense and respond to the mechanical properties of their microenvironment through specialized molecular systems that convert physical forces into biochemical signals. These processes involve integrin‐mediated adhesion complexes, cytoskeletal tension generated by actin‐myosin contractility, focal adhesion kinase signaling, and mechanosensitive ion channels such as Piezo1.^36^, ^37^ Mechanical cues transmitted through these structures activate intracellular signaling cascades that regulate gene expression programs associated with cell proliferation, differentiation, and ECM production.^38^, ^39^ Among these pathways, the Hippo signaling pathway and its downstream effectors YAP and transcriptional co‐activator with PDZ‐binding motif (TAZ) play a central role in translating mechanical stimuli into transcriptional responses.

In healthy tissues, the Hippo pathway functions as a regulatory system controlling organ size, tissue repair, and cellular homeostasis. Under conditions of increased matrix stiffness or altered mechanical stress^40^, YAP and TAZ translocate to the nucleus and interact with transcription factors such as TEAD, thereby promoting the expression of genes involved in cell proliferation, cytoskeletal remodeling, and ECM synthesis.^41^ Persistent activation of YAP/TAZ signaling has been implicated in fibrotic processes across multiple organs, including the liver, lung, heart, kidney, and skin.^41^, ^42^ For example, in liver fibrosis, YAP/TAZ activation contributes to HSC activation and excessive collagen production, whereas in pulmonary fibrosis, these transcriptional regulators promote fibroblast proliferation and resistance to apoptosis.^43^ Consequently, pharmacological strategies aimed at modulating YAP/TAZ activity, inhibiting upstream integrin signaling or altering cytoskeletal tension are being explored as potential antifibrotic interventions.

Another important mechanotransduction pathway involves integrin receptors, which mediate the physical linkage between cells and the ECM.^44^ Integrins transmit mechanical forces from the ECM to intracellular signaling complexes located at focal adhesions, thereby regulating cell migration, differentiation, and survival. In fibrotic tissues, certain integrin subtypes, such as αvβ6 and αvβ1, are known to activate latent TGF‐β, one of the most potent profibrotic cytokines.^45^ Targeting these integrins with selective inhibitors may therefore reduce both mechanical signaling and TGF‐β activation simultaneously. Several experimental compounds designed to block integrin‐mediated mechanotransduction have demonstrated antifibrotic effects in preclinical models, highlighting the therapeutic potential of targeting cell–matrix interactions directly.^45^, ^46^

Beyond modulating cellular signaling pathways, another emerging strategy focuses on normalizing the ECM itself. Fibrotic tissues are characterized not only by excessive deposition of collagen fibers but also by profound alterations in matrix architecture and composition. Increased crosslinking of collagen fibers leads to enhanced mechanical stiffness and decreased susceptibility to proteolytic degradation. Enzymes belonging to the lysyl oxidase (LOX) family, including LOX and LOX‐like proteins (LOXL1‐4), catalyze the oxidative deamination of lysine residues in collagen and elastin, thereby promoting covalent crosslink formation between matrix fibers.^47^, ^48^ Elevated expression of LOX enzymes has been observed in many fibrotic conditions and correlates with increased tissue stiffness and disease severity.

Pharmacological inhibition of LOX or LOXL enzymes therefore represents a promising strategy to restore matrix compliance and facilitate tissue remodeling. By reducing collagen crosslinking, these inhibitors may increase the susceptibility of fibrotic ECM to degradation and improve the mechanical properties of affected tissues. Experimental studies have shown that inhibition of LOX activity can reduce fibrosis progression in models of liver, lung, and cardiac fibrosis.^49^, ^50^, ^51^ Although clinical translation of LOX inhibitors has proven challenging due to issues related to specificity and systemic toxicity, continued research into selective inhibitors and targeted delivery approaches may overcome these limitations.

Complementary therapeutic approaches aim to enhance the natural turnover and degradation of ECM components. Under physiological conditions, ECM homeostasis is maintained through a balance between matrix synthesis and degradation mediated by proteolytic enzymes such as matrix metalloproteinases (MMPs) and their endogenous inhibitors, the tissue inhibitors of metalloproteinases (TIMPs).^52^ In fibrotic tissues, this balance becomes disrupted, leading to reduced matrix degradation and progressive accumulation of ECM proteins. Strategies designed to restore proteolytic activity, either by enhancing MMP function or modulating the MMP/TIMP ratio, may therefore promote the gradual resolution of fibrotic scars in many diseases.^52^, ^53^

In addition to extracellular proteolysis, intracellular pathways such as autophagy also contribute to matrix turnover and wound healing.^54^ Matrix constituents have been shown to regulate autophagy in both a positive (activators) or negative (inhibitors) manner.^55^ Autophagy plays an important role in cellular quality control and metabolic adaptation, and emerging evidence suggests that it may participate in the degradation of internalized collagen fragments and other matrix components.^55^ Enhancing autophagy‐mediated clearance pathways may therefore represent another mechanism for promoting ECM remodeling. Furthermore, advances in biomaterials and tissue engineering have opened new possibilities for designing therapeutic scaffolds capable of modulating the mechanical environment of damaged tissues and guiding regenerative processes.

Cell‐specific sensitization represents a further translational avenue aimed at directly targeting the cellular populations responsible for matrix production. MFs, which are major producers of collagen I in fibrotic tissues, arise predominantly from resident mesenchymal populations in many settings, whereas bone marrow‐derived fibrocytes, perivascular cells, epithelial plasticity, and endothelial‐to‐mesenchymal transition may contribute in an organ‐, injury‐, and model‐dependent manner.^56^, ^57^ Once activated, MFs exhibit enhanced contractility, increased secretion of ECM proteins, and resistance to apoptosis. In many fibrotic conditions, these cells persist even after the initial injurious stimulus is resolved, thereby maintaining the pathological remodeling process.

Therapeutic strategies that restore apoptotic sensitivity in activated MFs may accelerate fibrosis resolution. One approach involves targeting survival pathways that are abnormally activated in these cells, such as the PI3K/protein kinase B pathway or anti‐apoptotic members of the BCL‐2 family.^58^, ^59^, ^60^ Inhibition of these pathways can selectively induce apoptosis in activated fibroblasts while sparing quiescent cells, thereby reducing the population of matrix‐producing cells within fibrotic tissue.

Another promising strategy involves the elimination of senescent cells that accumulate during chronic tissue injury. Cellular senescence is characterized by permanent cell cycle arrest accompanied by the secretion of a complex mixture of cytokines, growth factors, and proteases collectively referred to as the senescence‐associated secretory phenotype.^61^ Senescent fibroblasts and other cell types can contribute to fibrosis by maintaining a chronic inflammatory and profibrotic microenvironment. Senolytic therapies (i.e., senolytics), which selectively eliminate senescent cells, have demonstrated encouraging antifibrotic effects in preclinical models of lung, liver, and kidney fibrosis.^62^, ^63^ By removing these dysfunctional cells, senolytic agents may reduce persistent inflammatory signaling and facilitate tissue repair.

In addition to pharmacological interventions, regenerative medicine approaches are also being explored to counteract fibrotic remodeling. MSCs and other progenitor cell populations have been investigated for their ability to modulate immune responses, inhibit fibroblast activation, and promote tissue regeneration.^64^, ^65^ These cells exert many of their therapeutic effects through the secretion of paracrine factors and EVs that influence surrounding cells and modulate the ECM environment. Although the clinical application of cell‐based therapies remains under active investigation, early studies suggest that they may represent a complementary strategy for restoring tissue homeostasis in chronic fibrotic diseases.^65^, ^66^

Together, these diverse therapeutic strategies illustrate a significant shift from traditional single‐pathway pharmacological approaches toward integrated interventions that address both the cellular drivers of fibrosis and the physical characteristics of the ECM. By simultaneously targeting mechanotransduction pathways, ECM composition and crosslinking, matrix degradation mechanisms, and the persistence of activated fibroblasts, translational mechanobiology aims to disrupt the core feedback mechanisms that sustain fibrotic disease.

Importantly, the development of effective antifibrotic therapies will likely require a systems‐level understanding of the complex interactions between cells, ECM components, and mechanical forces within tissues. Advances in imaging technologies, high‐resolution molecular profiling, and bioengineered tissue models are providing new opportunities to investigate these interactions in greater detail. Such approaches may help identify patient‐specific patterns of fibrotic remodeling and enable more precise targeting of therapeutic interventions. Ultimately, by integrating mechanistic insights from basic science with translational and clinical research, mechanobiology offers a promising framework for the development of next‐generation antifibrotic therapies. Interventions capable of restoring the dynamic equilibrium between cells and their extracellular environment may not only slow the progression of fibrosis but also promote genuine tissue regeneration and functional recovery across multiple organ systems.

## 6 STATE OF THE ART IN TREATMENT OF FIBROSIS

Treatment of fibrosis now targets biological pathways causing scarring rather than just inflammation. Most therapies aim to slow disease progression, and recently, new drugs have been approved for pulmonary fibrosis and noncirrhotic metabolic dysfunction‐associated steatohepatitis (MASH) with fibrosis.

### 6.1 Idiopathic pulmonary fibrosis

Nerandomilast is an orally administered thienopyrimidine sulfoxide that targets phosphodiesterase 4B (PDE4B) with high specificity and has shown efficacy in reducing FVC decline in a Phase 3 trial of patients with IPF. Its sulfoxide (S) stereochemistry and (5R) cyclobutyl configuration ensure a strong affinity and minimal effect on other PDE4 subtypes. In a Phase 3, double‐blind trial, 1177 patients with IPF were randomly assigned to receive either nerandomilast 18 mg or 9 mg twice daily or a placebo.^67^ After 52 weeks, the decline in FVC was smaller in both nerandomilast groups compared to the placebo, with adjusted differences of 68.8 mL (18 mg) and 44.9 mL (9 mg). Diarrhea was the most common side effect, occurring more frequently in the nerandomilast groups. Serious adverse events were similar across all groups. The study concluded that nerandomilast reduced FVC decline compared to the placebo.

### 6.2 Hepatic fibrosis

Hepatic fibrosis, which results from chronic hepatotoxic or cholestatic injuries, chronic viral infections (HBV and hepatitis C virus), alcohol, or MASLD, may be partially reversible, particularly in early stages, if the underlying cause of injury is effectively removed. This is of special interest for systemic health given the pivotal role of liver fibrosis in gauging the risks of hepatic and extrahepatic outcomes.^68^, ^69^

Resmetirom, approved for liver fibrosis associated with noncirrhotic non‐alcoholic steatohepatitis (NASH), acts as a thyroid receptor beta agonist to reduce hepatic fat and improve liver stiffness. Evidence for the efficacy of this innovative drug was primarily provided by the pivotal Phase 3 MAESTRO‐NASH clinical trial (NCT03900429), a double‐blind, randomized, placebo‐controlled study involving 966 adults with biopsy‐proven noncirrhotic NASH and fibrosis stages F1B, F2, or F3.^70^ After 52 weeks, the trial showed that resmetirom led to NASH resolution in 25.9% (80 mg) and 29.9% (100 mg) of patients, compared to 9.7% for placebo. Fibrosis improved by at least one stage in 24.2% (80 mg) and 25.9% (100 mg) versus 14.2% for placebo. Liver fat was reduced by up to 46.6%. Low‐density lipoprotein cholesterol dropped by 13.6%–16.3%, along with other lipids. In March 2024, the U.S. food and drug administration gave accelerated approval to resmetirom as the first treatment for adults with noncirrhotic NASH and moderate‐to‐advanced liver fibrosis.

Semaglutide has received accelerated or conditional approval in the United States for the treatment of adults with MASH who have moderate‐to‐advanced (stage F2–F3) liver fibrosis based on Phase 3 ESSENCE trial interim results showing that 37% of patients on semaglutide 2.4 mg achieved at least a 1‐stage improvement in fibrosis without worsening of MASH, compared to 22% in the placebo group.^71^ Semaglutide's antifibrotic effect mainly occurs indirectly through significant weight loss and better metabolic markers rather than directly affecting liver stellate cells.^72^ Currently, this drug is not approved for patients with cirrhosis (i.e., fibrosis stage F4) and semaglutide did not significantly improve fibrosis or achievement of NASH resolution versus placebo in compensated cirrhosis.^73^

### 6.3 Dermal fibrosis

Fibrosis plays a critical role in the formation of keloids and hypertrophic scars that develop after burns, radiation, or skin injuries. Dermal fibrosis is also a significant factor in autoimmune diseases such as scleroderma.^74^ The typical treatment for dermal lesions includes the use of locally administered steroidal anti‐inflammatory agents, as well as nonpharmacological options such as cryotherapy, excision, irradiation, or fat grafting.^75^ However, these methods are often insufficient, particularly for fibrotic disorders related to autoimmune conditions. As a result, there is ongoing research into new antifibrotic treatments, which are discussed in more detail elsewhere.^74^

### 6.4 Cardiac fibrosis

Fibrosis frequently occurs in cardiac diseases, such as myocardial infarction, cardiac hypertrophy due to pressure or volume overload, and dilated cardiomyopathy related to diabetes or other conditions.^76^ Cardiac fibrosis manifests in three main forms: replacement fibrosis (scar tissue replaces lost cardiomyocytes after myocardial infarction), interstitial fibrosis (ECM expands within muscle layers), and perivascular fibrosis (ECM accumulates around micro‐vessels without major cell loss).^77^ Interstitial and perivascular fibrosis types result from sustained fibrogenic activation.^77^ Most drugs investigated for cardiac fibrosis often lower blood pressure and are studied within chronic cardiomyopathy contexts, as reviewed elsewhere.^74^

### 6.5 Renal fibrosis

Renal fibrosis, a common occurrence in chronic kidney disease, impacts the glomeruli, tubulointerstitium, and vasculature. It can result in decrease in kidney function, potentially advancing to end‐stage renal disease, necessitating renal replacement therapy, or leading to death.^78^ Some experimental and clinical evidence suggests that certain aspects of renal fibrosis may be partially reversible under specific conditions, particularly when the underlying injury is effectively treated.^79^ The most frequently prescribed treatments include modulators of the renin–angiotensin–aldosterone axis to lower intraglomerular pressure, along with glucose‐lowering medications if diabetes is a contributing factor.^80^ Although these drugs may possess direct antifibrotic properties,^81^, ^82^ the clinical significance of their antifibrotic effects is still not clearly defined. Other medications currently in clinical trials have been discussed in previous reviews.^74^

### 6.6 Existing treatment challenges and emerging therapeutic strategies

Approved drugs for fibrosis can only slow disease progression; they do not reliably halt or reverse scarring. Many have significant side effects, causing some patients to stop treatment. Organ transplantation is the only cure for severe cases but is limited by donor shortages. Research now focuses on repairing tissue damage using MSCs and induced pluripotent stem cells to restore tissues and control immune responses in regenerative medicine.^83^ In some cases, artesunate, an antimalarial agent, may be repurposed as effective antifibrotics by targeting the MD2/TLR4 signaling pathway in cardiac fibroblasts.^84^ Finally, fibroblast activation protein inhibitor positron emission tomography imaging is being utilized to identify activated fibroblasts prior to the onset of visible scarring on conventional scans, thereby enabling earlier intervention.^85^

## 7 CONCLUSION AND RESEARCH AGENDA

Fibrosis is a common pathological endpoint of chronic injury in various organs, driven by complex interactions between cellular signaling networks and the ECM. Increasing evidence shows that the matrix is not just a passive structural scaffold but also an active regulator of cellular behavior through biochemical and mechanical cues. Mechanotransduction pathways, paratensile signaling, and intercellular communication through cytokines and EVs establish a self‐reinforcing feedback loop that sustains fibroblast activation and matrix deposition.^8^

Recent advances in mechanobiology have shed light on how these processes work in different organ systems, revealing conserved signaling pathways such as integrin‐mediated mechanosensing and the Hippo‐YAP/TAZ axis. Quantitative imaging techniques and digital pathology tools offer new ways to continuously measure fibrosis, moving away from coarse staging systems. These advancements are changing the way we view fibrotic diseases, focusing on early detection of pathological remodeling and identifying actionable therapeutic targets. Despite these advances, challenges remain. Understanding the precise mechanisms linking mechanical signals in the matrix to transcriptional programs in different cell populations requires further study. The heterogeneous nature of fibrosis across organs and disease causes suggests that effective therapies may need organ‐specific adaptations. Translating experimental mechanobiological insights into clinical treatments is difficult due to the complexity of modeling the intricate mechanical environment of living tissues.

A translational mechanobiology approach requires moving from descriptive fibrosis staging toward measurable decision points that reflect the activity and reversibility of the fibrotic process. Future studies should therefore determine which combinations of matrix stiffness, collagen architecture, molecular fibroblast activation, imaging biomarkers, and functional decline identify patients who are most likely to benefit from antifibrotic intervention. Such thresholds should be validated separately as diagnostic thresholds, treatment‐action thresholds, and response thresholds because each serves a different clinical purpose. This approach may allow earlier intervention, more rational trial design, and better distinction between slowed progression and true tissue remodeling. There should be a focus on research that prioritize interdisciplinary approaches that combine cell biology, bioengineering, imaging science, and clinical investigation (Table 3).

**TABLE 3:** Research agenda in management of organ fibrosis.^35^, ^84^, ^85^, ^86^, ^87^, ^88^, ^89^, ^90^, ^91^, ^92^, ^93^, ^94^, ^95^, ^96^, ^97^

	References
Key research priorities	
*Elucidating fibroblast heterogeneity*: Gain insights into the various subtypes of fibroblasts and their functional roles across distinct tissues, with the aim of identifying and targeting the cells primarily responsible for the progression of fibrosis	^86^, ^87^
*Macrophage modulation*: Target macrophage recruitment and polarization to mitigate the wound‐healing phenotypes associated with excessive formation of fibrotic tissue	^88^
*Inhibition of necroptosis*: Examine necroptotic pathways, which contribute to inflammatory cell death and exacerbate fibrosis, as a potential therapeutic target	^89^
*Senescence and aging*: Examine the relationship between cellular senescence and the development of tissue fibrosis	^90^
*Mechanotransduction*: Investigate cellular mechanisms for sensing and responding to ECM stiffness, with the aim of advancing therapeutic strategies to counteract tissue stiffening	^35^
Future therapeutic strategies	
*Combination therapies*: Development, evaluation, and application of multi‐drug regimens, such as the concurrent use of antifibrotics including nintedanib and pirfenidone, that address multiple biological pathways simultaneously	^91^
*Precision medicine approaches*: Leverage single‐cell RNA sequencing, spatial transcriptomics, and genetic data to determine individualized therapeutic targets for patients. Special emphasis on the determinants of fibrosis in men and women	^92^, ^93^
*Targeting macrophages and TGF‐β*: Employ monocyte‐derived macrophage depletion via anti‐CSF1R antibodies, together with the modulation of TGF‐β pathways, to mitigate fibrogenesis	^94^
*Artificial intelligence (AI)*: Use AI to expedite drug discovery, including the identification of novel kinase inhibitors such as rentosertib	^95^
*Cell‐based therapies*: Develop CAR‐T cell strategies for the treatment of cardiac fibrosis and apply mesenchymal stem cells (MSCs) for regenerative medicine purposes	^96^
Diagnostic and monitoring research	
*Noninvasive biomarkers*: Enhance the application of multiorgan MRI with T1 mapping for the assessment and ongoing monitoring of fibrosis severity, eliminating the need for biopsy procedures	^97^
*Clinical trial endpoints*: Establish well‐defined, clinically meaningful endpoints in clinical studies that assess both structural changes and functional improvements, especially in trials involving multiple organs	^31^
Nonpharmacological and holistic care	
*Exercise protocols*: Examine the most effective forms and intensities of physical activity for lowering inflammatory cytokines and fibrosis indicators	^98^
*Integrated care models*: Implement inclusive and thorough approaches for patient management, encompassing psychosocial support, nutritional guidance, and the initiation of palliative care at an early stage for individuals with severe conditions	^99^

Abbreviations: ECM, extracellular matrix; LOX, lysyl oxidase; MRI, magnetic resonance imaging; TGF‐β, transforming growth factor‐β.

Improved in vitro models, such as organ‐on‐chip systems and engineered matrices with adjustable stiffness, can better replicate the mechanical microenvironment of fibrotic tissues. Longitudinal imaging and molecular biomarkers should be integrated into clinical studies to identify early signs of therapeutic responses. Targeting both the biochemical and physical aspects of fibrosis holds promise for disrupting the pathological feedback loop driving the disease. By combining mechanistic insights with diagnostic tools and innovative therapies, translational mechanobiology offers a path to more effective antifibrotic treatments. Ongoing research in this field has the potential to produce interventions that can not only halt the progression of fibrosis but also restore normal tissue architecture and function in different organs. Furthermore, these advancements could enable the prompt initiation of palliative care for patients with severe conditions.

## AUTHOR CONTRIBUTIONS

Amedeo Lonardo and Ralf Weiskirchen conceptualized the study, curated the results, and finalized the manuscript.

## CONFLICT OF INTEREST STATEMENT

R.W. is the Executive Editor of *Journal of Cell Communication and Signaling* and Editor of the Special Issue “Cell‐Cell and Extracellular Matrix Signaling in Multiorgan Fibrosis: Mechanisms, Quantitative Therapeutic Thresholds, and Translational Targeting for Tissue Remodeling.” A.L. has nothing to declare.

## ETHICS STATEMENT

Not applicable.

## Data Availability

Data sharing not applicable to this article as no datasets were generated or analyzed during the current study.
